# Laboratory Investigation of the Low-Temperature Crack Resistance of Wood Tar-Based Rejuvenated Asphalt Mixture Based on the Semi-Circular Bend and Trabecular Bending Test

**DOI:** 10.3390/ma15207223

**Published:** 2022-10-17

**Authors:** Kefei Liu, Chonglin Liu, Quan Li, Kang Jiang

**Affiliations:** 1 School of Civil Engineering, Central South University of Forestry & Technology, Changsha 410004, China; 2 Hunan Provincial Engineering Research Center for Construction Solid Wastes Recycling, Changsha 410205, China; 3 Hunan Communications Research Institute Co., Ltd., Changsha 410015, China

**Keywords:** wood tar-based rejuvenated asphalt, low-temperature crack resistance, trabecular bending creep test, creep damage model

## Abstract

Low-temperature crack resistance is the core issue affecting the promotion of rejuvenated asphalt, but most current studies do not consider the creep relaxation characteristics of rejuvenated asphalt mixture at low temperatures, which is inconsistent with the actual situation. To explore the low-temperature crack resistance of a wood tar-based rejuvenated asphalt mixture, we observed the low-temperature crack resistance of styrene butadiene styrene (SBS) modified asphalt, wood tar-based rejuvenated asphalt, and RA-102 rejuvenated asphalt and their mixtures using laboratory tests. Our results showed that the low temperature crack resistance of the wood tar-based rejuvenated asphalt mixture was better than that of the RA-102 rejuvenated asphalt mixture, but slightly worse than that of the original SBS asphalt mixture. After the synergistic action of wood tar and biomass fiber, wood tar can be fully mixed into the new asphalt, effectively alleviating the bonding failure between asphalt and aggregate and improving the stiffness of the mixture, so that the toughness and crack resistance of rejuvenated asphalt mixture at low temperatures are evidently improved. Wood tar-based rejuvenated asphalt mixture has a good creep deformation ability at low temperatures. The established creep damage model can better describe the flexural creep performance of rejuvenated asphalt mixtures at low temperatures, and can be used to infer the deformation characteristics at other low temperatures.

## 1. Introduction

Low-temperature cracking of asphalt pavement is a universal phenomenon, and is one of the main forms of pavement damage [1]. When the temperature drops sharply or the temperature difference becomes too large, the asphalt mixture will produce micro-cracks due to the temperature stress. Micro-cracks can widen or develop into more cracks during seasonal alternation and repeated temperature changes, which will directly affect the service performance and life of the pavement. If rainwater, silt, and other impurities penetrate into the base through the cracks, it weakens the strength of the base or soil, decreasing the bearing capacity of the pavement, and creating a vicious cycle that accelerates damage of pavement [2].

Low-temperature crack resistance is the core issue affecting the promotion of rejuvenated asphalt. Scholars at home and abroad have used various methods to analyze the performance decay process of asphalt mixtures at low temperature and the influence of different kinds of asphalt on low-temperature performance. Rodland et al. [3] found that particles from tire and road wear constitute the largest source of microplastic particles into the environment, and Choi et al. [4] confirmed that using tire-derived fuel (TDF) fly ash as filler had good mechanical properties in the hot mix asphalt mixture. Yin et al. [5] found that improving the flexural strength of the asphalt mixture could also improve its fracture toughness. Zhang et al. [6] used the semicircular bending test (SCB) of the fracture energy evaluation index to study influencing factors on the low-temperature crack resistance of a rejuvenated asphalt mixture. Hao et al. [7] compared the changes in flexural strain of a rejuvenated asphalt mixture under different freeze–thaw cycles, and found that with an increase in freeze–thaw cycles, the flexural strain of the rejuvenated asphalt mixture decreased. Wang et al. [8] established a creep damage model and six-element generalized Maxwell model through a flexural creep test and direct tensile stress relaxation test. It was found that the low-temperature crack resistance of a polyphosphoric acid (PPA)- styrene butadiene styrene (SBS) composite modified asphalt mixture was better than that of a SBS modified asphalt. Li et al. [9] found through a trabecular low-temperature bending test that the asphalt mixture modified by a composite rubber powder had better low-temperature deformation ability compared with the original asphalt. Asphalt rejuvenation is the reverse process of aging. The relative content of asphaltene can be reduced by adding rejuvenator to aged asphalt, and the penetration and ductility of asphalt is improved to restore its service performance. Current research on the low-temperature crack resistance of asphalt mixtures mostly focuses on the original asphalt mixture and less attention is paid to rejuvenated asphalt mixtures. Most of the research methods and indexes on low-temperature crack resistance of asphalt mixtures are based on strength and deformation theory, without considering the creep relaxation characteristics of rejuvenated asphalt mixtures at low temperature, which is inconsistent with the actual situation.

As a high-temperature pyrolysis product of bamboo/wood biomass material, wood tar has the characteristics of being a wide source, waste-utilizing, environmentally friendly, and regenerative resource [10]. Studies have shown that wood tar can effectively improve the fatigue performance and low-temperature crack resistance of asphalt when used as an asphalt modifier [11]. When used as aged asphalt rejuvenator, wood tar can decrease the viscosity of aged asphalt and increase its penetration. The road performance of wood tar-based rejuvenated asphalt meets specification requirements, and its high-temperature deformation resistance is better than that of the original asphalt matrix [12]. In this paper, wood tar, a widely distributed and widely used bamboo pyrolysis product, was used as the basic raw material to mix with biomass fiber, plasticizer, compatibilizer, and stabilizer to prepare a composite rejuvenator. Taking SBS-modified original asphalt and commercial RA-102 rejuvenated asphalt as control groups, the low-temperature properties of the three types of asphalt were measured by a bending beam rheometer (BBR) test. The corresponding asphalt mixtures were prepared. Through the SCB test, trabecular bending test, and trabecular bending creep test, the low-temperature rheological properties of the wood tar-based rejuvenated asphalt mixture were studied at different temperatures, using rheological indexes, such as creep rate, fracture energy density, and flexural tensile strain. A creep damage model was established to evaluate the low-temperature crack resistance of wood tar-based rejuvenated asphalt mixture. Based on the above laboratory tests, this paper aims to:

(1) Establish a damage creep model to describe the bending creep properties of a rejuvenated asphalt mixture at low temperatures based on the creep relaxation characteristics and laboratory test results of rejuvenated asphalt mixture at low temperatures.
(2) Comprehensively evaluate the road performance of wood tar-based rejuvenated asphalt and promote the practical application of wood tar-based rejuvenated asphalt.

## 2. Materials and Methods

### 2.1. Base Materials

#### 2.1.1. Asphalt

In this study, SBS-modified asphalt was used as the original asphalt, and its basic properties are shown in Table 1. Penetration is the depth of the standard cone (weighing 150 g) that sinks into an asphalt sample insulated at 25 °C within 5 s; penetration index is an index describing the temperature sensitivity of asphalt—the higher the penetration index, the lower the temperature sensitivity of asphalt; ductility refers to the extension of asphalt; viscosity is the resistance of asphalt to flow; and softening point refers to the temperature at which the asphalt sample is softened by heat and sags.

#### 2.1.2. Rejuvenator

Wood tar-based asphalt rejuvenator was prepared using wood tar, biomass fiber, plasticizer, compatibilizer, and stabilizer as its basic components. Among them, wood tar was produced in an environmental charcoal factory in Youxian County (Zhuzhou, China) and its raw material was bamboo. Biomass fiber is a kind of modified flocculent fiber made from the stem or bark of bamboo. The basic properties of the wood tar and biomass fiber are shown in Table 2. Density of the wood tar was determined by a petroleum densitometer (Hongtuo, Dongguan, China), the pH value of wood tar was measured by an industrial pH meter, the density of bamboo fiber was determined by the specific gravity flask method, and the length of bamboo fiber was measured by a combing method. The plasticizer (dioctyl phthalate), stabilizer (lauryl propylene diamine), and compatibilizer (maleic anhydride) were all purchased from Jirui Chemical Equipment Co., Ltd. (Yueyang, China), and were analytically pure.

For the comparative study, RA-102 rejuvenator (main component: extract oil) provided by Subbott New Materials Co., Ltd. (Nanjing, China) was used as the reference rejuvenator. The basic properties of the two rejuvenators are shown in Table 3. Among them, flash point refers to the lowest temperature at which a mixture of material and external air will flash and burn immediately when it comes into contact with a flame; viscosity ratio before and after RTFOT (Rolling thin film oven test) refers to the ratio of the viscosity of asphalt after RTFOT aging to the viscosity before aging; and mass change before and after RTFOT refers to the ratio of the mass of asphalt after RTFOT aging to the viscosity before aging.

### 2.2. Preparation of Rejuvenated Asphalt

**Figure 1:**
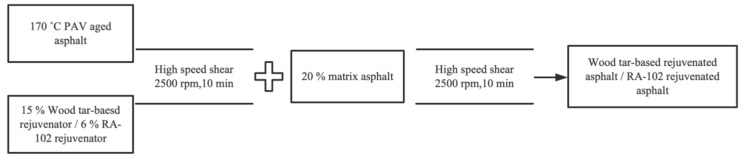
Preparation process of rejuvenated asphalt.

The original asphalt was subjected to a thin film oven test (TFOT) and pressure aging vessel (PAV) to prepare aged asphalt. The test conditions of TFOT was 163 °C, being aged for 5 h, and the test conditions of PAV was 100 °C at 2.1 Mpa, aging for 20 h. The PAV-aged asphalt was treated according to the process shown in Figure 1 to prepare wood tar-based rejuvenated asphalt and RA-102 rejuvenated asphalt, respectively. According to previous research results, the wood tar-based rejuvenator and RA-102 rejuvenator were added to the aged asphalt at a mass ratio of 15 and 6%, respectively.

### 2.3. Aggregrates

Adopting basalt as the coarse aggregate of mixture, basalt chips as the fine aggregate of mixture, and limestone powder as the filler, all aggregates were produced in Hengyang Quarry (Hengyang, China). The basic technical indexes of each aggregate are shown in Table 4, Table 5 and Table 6. All technical performance of aggregates met the requirements of specification from the Technical Specification for Construction of Highway Asphalt Pavements (JTG F40-2017).

### 2.4. Mix Design of Asphalt Mixture

**Figure 2:**
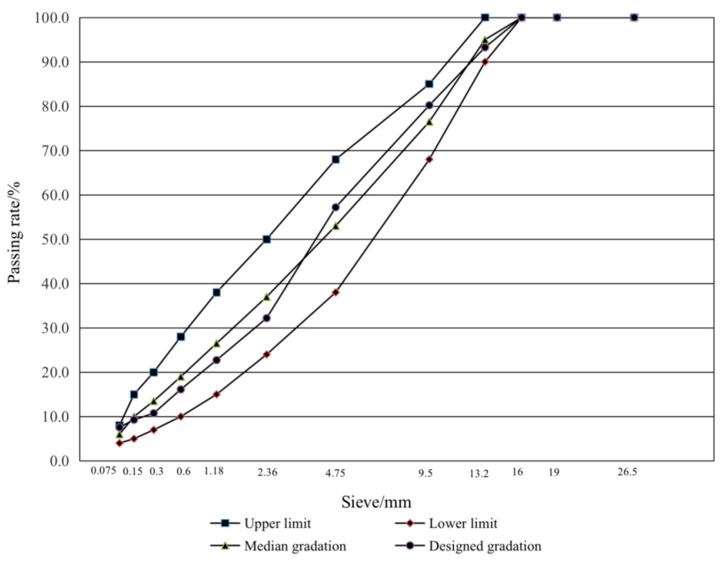
Synthesis gradation curve of AC-13.

In this paper, AC-13 gradation was used to prepare the asphalt mixture, and its synthetic gradation curve is shown in Figure 2. According to the Fuller maximum density curve theory, the gradation of the asphalt mixture fluctuated within a certain range, and the density curve index *n* was taken between 0.3–0.7. When *n* is equal to 0.7, the gradation range of the mixture is at the lower limit; when *n* is equal to 0.3, the gradation range of the mixture is at the upper limit. When *n* is equal to 0.45 (median gradation in Figure 2), the density of the mixture is the maximum. In this study, to control the passing rate of nominal maximum particle size between 90–95% and make the mixture form a good skeleton structure, the designed gradation in Figure 2 was used as the gradation curve of the asphalt mixture.

Based on the design porosity of 4%, the optimum asphalt content of original asphalt mixture, wood tar-based rejuvenated asphalt mixture, and RA-102 rejuvenated asphalt mixture were determined by the Marshall test to be 5.0, 6.0, and 5.6 wt%, respectively.

### 2.5. Experiments

#### 2.5.1. BBR Test

The BBR test was used to evaluate the low-temperature crack resistance of each asphalt binder. According to the T0627-2011 test method in the Standard Test Methods of Bitumen and Bituminous Mixtures for Highway Engineering (JTG E20-2011), the bending creep modulus *S* and creep rate *m* of the original asphalt, aged asphalt, and two rejuvenated asphalts were measured within the loading time of 8–240 s. The sample size was 127.00 mm × 6.35 mm × 12.70 mm. According to specification AASHTO T 312-2004, when the *S* value of asphalt is greater than 300 MPa or *m* value is less than 0.300, the low-temperature cracking resistance of asphalt binder cannot meet the requirements of specification. Based on previous research results [13], the test temperature of each asphalt binder were −6, −12, and −18 °C, respectively.

#### 2.5.2. SCB Test

**Figure 3:**
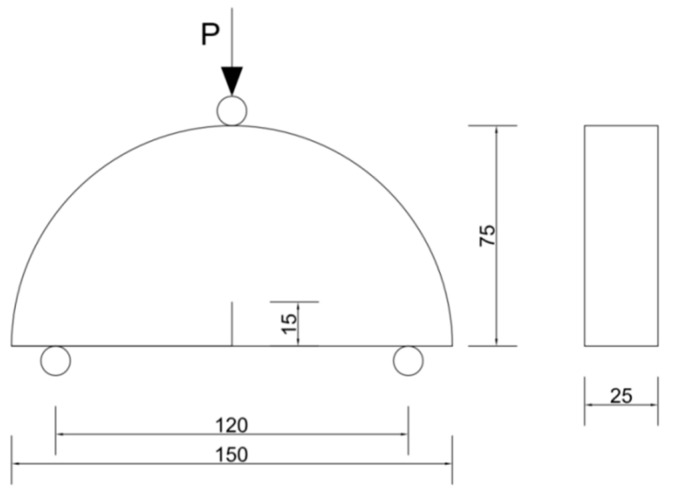
Semicircle bending test specimen (unit: mm).

To reduce the influence of permanent deformation caused by load, the SCB test was used to evaluate the low-temperature crack resistance of the asphalt mixture. According to specification AASHTO T 394-21, the cylindrical specimen of *φ*150 × 110 mm was prepared by a rotary compactometer (Earth Products, Hong Kong, China). The specimen was cut into *φ*150 × 25 mm disks, and then cut into two identical standard semicircle specimens. A straight slit with a depth of 15 mm and a width of 2.5 mm was cut along the midpoint of each semicircle specimen towards the semicircle direction (shown in Figure 3) [14]. The SCB test was conducted on the HYD-25 UTM tester (IPC Global, Melbourne, Australia), with a loading rate of 5 mm/min, bearing spacing of 120 mm, and test temperature of −12 °C. The flexural tensile stress *σ*_B_, flexural tensile strain *ε*_B_, and fracture energy density $\frac{dw}{dv}$ of the specimens during the flexural tensile failure test were used as evaluation indices to evaluate the low-temperature crack resistance of each asphalt mixture. The fracture energy density of the SCB specimen refers to the area surrounded by the stress–strain curve and the strain axis before the stress peak in the stress–strain curve obtained from the SCB test. Fracture energy density $\frac{dw}{dv}$ was determined based on the Equation (1) [15].

(1) $$\frac{dw}{dv}=\int_{o}^{\varepsilon_{0}}\sigma d\varepsilon$$

where, $\frac{dw}{dv}$ is the fracture energy density, kPa, and *ε*_0_ is the strain value corresponding to the peak stress in the stress–strain curve.

Combined with Figure 3, in the parameters on the left side of Equation (1) [14]: *w* is the fracture energy, N/m; *v* is the volume of the specimen, m^3^; and *v* = (*r* − *a*) × *t*, where *r* is the radius of the specimen (75 mm), *a* is the incision length (15 mm), and *t* is the thickness of the specimen (25 mm).

#### 2.5.3. Trabecular Bending Test

**Figure 4:**
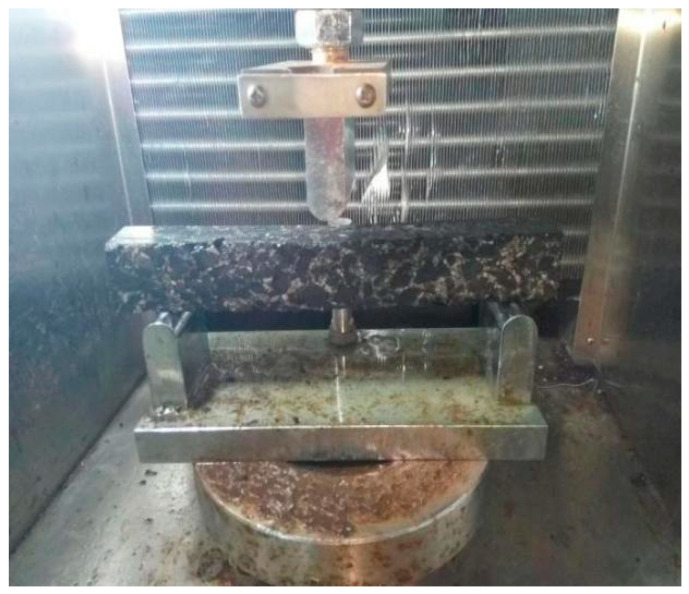
Low-temperature bending test device.

Considering the creep characteristics of the rejuvenated asphalt mixture at low temperatures, a trabecular bending creep test was used to test the crack resistance of the asphalt mixtures at low temperatures. According to the T0715-2011 asphalt mixture bending test method in specification JTG E20-2011, the flexural tensile strength *R*_B_, maximum flexural tensile strain *ε*_B_ at the bottom of the beam, and flexural stiffness modulus *S*_B_ of the specimen at failure were measured. The size of the specimen was the prism trabecula with a length of 250 ± 2.0 mm, width of 30 ± 2.0 mm, height of 35 ± 2.0 mm, and a span of 200 ± 0.5 mm. Test temperatures were −10, 0, and 10 °C, and the loading rate was 50 mm/min. The test device (Yaxing, Xi’an, China) is shown in Figure 4.

#### 2.5.4. Trabecular Bending Creep Test

Based on the trabecular bending test results, 10% of the failure load of each mixture was used as the load P0 for the bending creep test, and the change curve of displacement and time of asphalt mixture were measured, according to the T0728-2000 asphalt mixture bending creep test method in specification JTG E20-2011. The test temperatures were −10, 0, and 10 °C, the test frequency was 100 Hz, and loading time was 1 h.

## 3. Results and Discussion

### 3.1. BBR Test Result

#### 3.1.1. Creep Stiffness and Creep Rate

**Figure 5:**
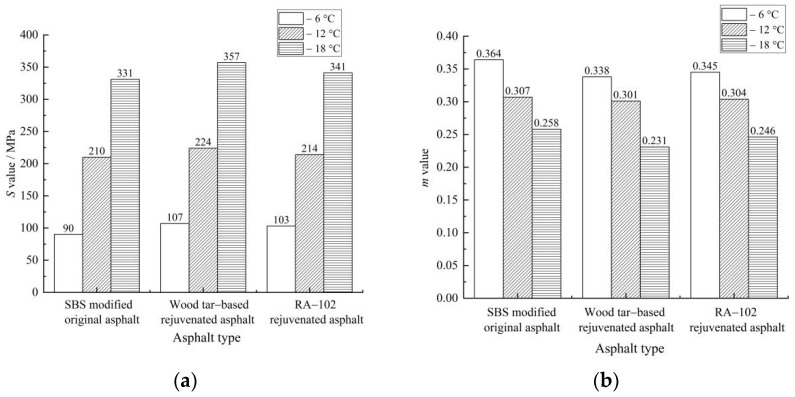
Test results of creep stiffness and creep rate of each asphalt binder: (**a**) *S* value and (**b**) *m* value.

The test results of creep stiffness *S* and creep rate *m* for each asphalt binder are shown in Figure 5.

As shown in Figure 5, the *S* value of asphalt significantly increased with a decrease in temperature, whereas the trend of the *m* value was completely opposite. The addition of rejuvenator can effectively restore the creep stiffness and creep rate of aged asphalt. When the test temperature was −12 °C, the *S* value of wood tar-based rejuvenated asphalt was 6.67 and 4.67% higher than that of the SBS-modified original asphalt and RA-102 rejuvenated asphalt, and the *m* value was 0.006 and 0.003 lower than the two, indicating that the low-temperature crack resistance of wood tar-based rejuvenated asphalt was slightly worse than that of RA-102 rejuvenated asphalt.

Essentially, the lower creep stiffness means that, when temperature drops, the temperature stress in the asphalt pavement is smaller, and the risk of pavement cracking is lower; the higher creep rate means that asphalt pavement has better stress relaxation ability [16]. Compared with original asphalt, wood tar-based rejuvenated asphalt has lower creep stiffness and higher creep rate. When temperature decreases and the pavement produces the same shrinkage strain, the temperature stress in the rejuvenated asphalt is larger than that of original asphalt, and its stress relaxation ability is lower. Consequently, rejuvenator can improve the low-temperature crack resistance of aged asphalt, but it cannot completely restore it to the level of original asphalt.

#### 3.1.2. Continuous Low-Temperature Classification Temperature

**Figure 6:**
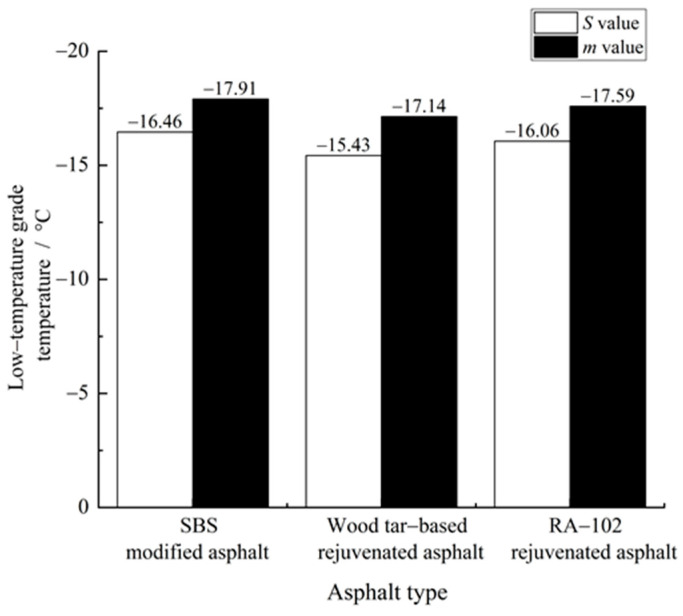
Calculation results of continuous low-temperature classification temperature of each asphalt.

Taking *S* = 300 Mpa and *m* = 0.300 as the low-temperature classification standard, the continuous low-temperature classification temperature of each asphalt was calculated, respectively [17], and the results are shown in Figure 6. As shown in Figure 6, the continuous classification temperature calculated from creep rate is lower than that calculated from creep stiffness. When the creep stiffness was used as the calculation index, the low-temperature classification temperature of wood tar-based rejuvenated asphalt was 1.03 and 0.63 °C higher than that of the SBS-modified original asphalt and RA-102 rejuvenated asphalt, respectively; when the creep rate was used as the calculation index, the corresponding increases were 0.77 and 0.45 °C, respectively. Therefore, the low-temperature classification of wood tar-based rejuvenated asphalt was basically in the same grade with SBS-modified original asphalt and RA-102 rejuvenated asphalt.

Based on the premise that the low-temperature crack resistance of the two recycled asphalt binders is comparable, the main raw materials of wood tar-based regenerating agent were biomass materials, and its material source and preparation were more in line with the demand of non-renewable resources conservation and sustainable development. Therefore, the low-temperature crack resistance of wood tar-based recycled asphalt mixture should be studied in-depth.

### 3.2. SCB Test Results

The SCB test results for each asphalt mixture are shown in Table 7.

As shown in Table 7 that the low-temperature flexural tensile stress and flexural tensile strain of the original asphalt mixture were obviously better than that of rejuvenated asphalt. The *σ*_B_ value of wood tar-based rejuvenated asphalt mixture was 6.24% lower than that of the SBS-modified original asphalt mixture and 12.93% higher than that of the RA-102 rejuvenated asphalt mixture, and its *ε*_B_ value was 5.85% lower than that of SBS-modified original asphalt mixture. This shows that the low-temperature crack resistance of the wood tar-based rejuvenated asphalt mixture was obviously better than that of the RA-102 rejuvenated asphalt mixture, but slightly worse than the original asphalt mixture. In addition, the fracture energy density of each asphalt mixture has the same law with its flexural tensile stress and flexural tensile strain, that is, the low-temperature crack resistance of each asphalt mixture is ranked as SBS-modified original asphalt mixture > wood tar-based rejuvenated asphalt mixture > RA-102 rejuvenated asphalt mixture. The reason is that the original asphalt has good fluidity and larger deformation under the same load, so it has a large flexural tensile strain and fracture energy density. The good low-temperature crack resistance of wood tar-based rejuvenated asphalt mixture comes from the synergy of wood tar and biomass fiber. They can fully infiltrate the new asphalt, effectively alleviating bond failure between asphalt and aggregates, improving the stiffness of the mixture, and significantly improving the toughness and crack resistance of the rejuvenated asphalt mixture at low temperatures [18]. RA-102 rejuvenated asphalt is thick and hard, and its toughness and fluidity at low temperatures are poor, so its flexural tensile strain and fracture energy density are small.

**Figure 7:**
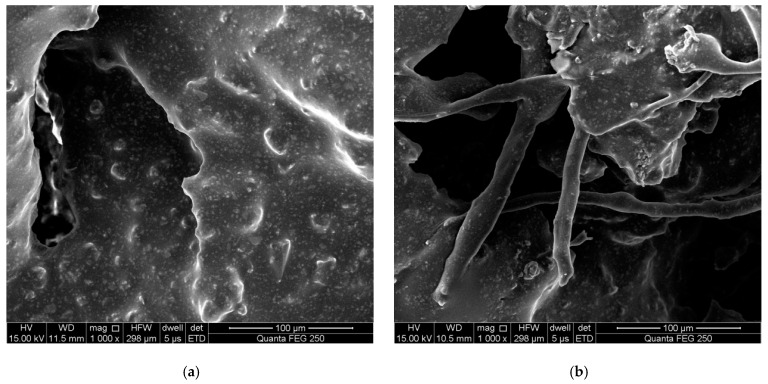
Failure interface of SCB test of different asphalt mixtures: (**a**) non-fiber (×1000) and (**b**) biomass fiber-reinforced (×1000).

In summary, the low-temperature crack resistance of wood tar-based rejuvenated asphalt was slightly worse than that of SBS-modified original asphalt and RA-102 rejuvenated asphalt, whereas the low-temperature crack resistance of this mixture was weaker than that of the SBS-modified original asphalt mixture, but better than that of the RA-102 rejuvenated asphalt mixture. This is related to the biomass fiber contained in the wood tar-based rejuvenator. Microscopic test results (shown in Figure 7) showed that there was a large amount of structural asphalt adsorbed on the fiber surface, and its strong coating force could effectively improve the interface connection between asphalt and aggregate, thus delaying the development of cracks at low temperatures. In addition, biomass fibers are slender filaments, just like the steel bars in reinforced concrete, and the whole mixture is connected through the fiber network. They may improve tensile properties at low temperatures to maintain the integrity of the whole structure [19], thus increasing crack resistance of the asphalt mixture.

### 3.3. Trabecular Bending Test

**Figure 8:**
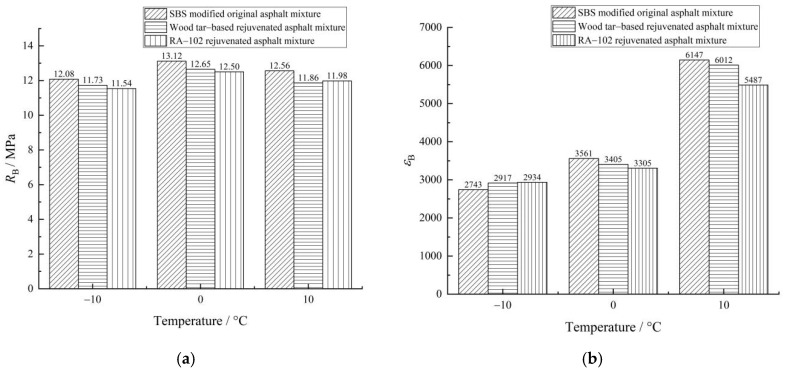

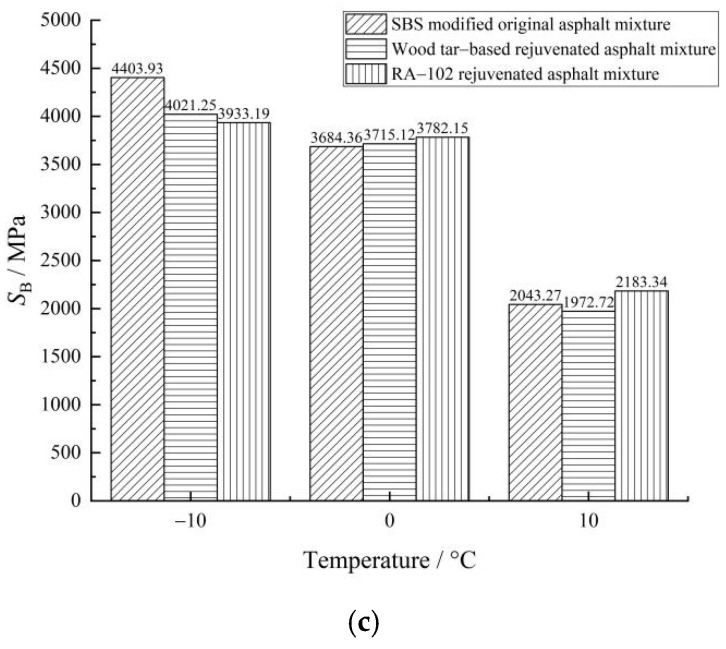
Trabecular bending test results of each asphalt mixture: (**a**) flexural tensile strength, (**b**) flexural tensile strain, and (**c**) stiffness modulus.

The trabecular bending test can provide important mechanical parameters for pavement design and predict the cracking of asphalt pavement. The test results are of great importance for the practical application of pavement material. The flexural tensile strength can characterize the tensile strength of asphalt mixture at low temperatures, whereas the flexural tensile strain reflects the elastic–plastic characteristics of the asphalt mixture at low temperatures. The stiffness modulus is the ratio of flexural tensile strength to flexural tensile strain, which can reflect the flexibility of the asphalt mixture as a whole [20]. The smaller the stiffness modulus, the better the flexibility of asphalt mixture and the better the low-temperature crack resistance. The trabecular bending test results of each asphalt mixture are shown in Figure 8.

As shown in Figure 8a, at −10–0 °C, the flexural tensile strength of each asphalt mixture increases with an increase in temperature; at 0–10 °C, the trend is quite the opposite, i.e., 0 °C is the turning point. This is because during the process of temperature change from −10 °C to 10 °C, the brittle failure of the asphalt mixture gradually changes to ductile failure. The increase in temperature enhances the viscosity of asphalt and changes the dominant characteristics of viscoelastic properties of the asphalt mixture [21]. At different temperatures, the flexural tensile strength of the wood tar-based rejuvenated asphalt mixture is higher than that of the RA-102 rejuvenated asphalt mixture, indicating that a wood tar-based rejuvenator has a better recovery effect on the low-temperature performance of aged asphalt mixtures.

As shown in Figure 8b,c, the flexural tensile strain of each asphalt mixture gradually increased with the increase in temperature, with the increase in the range of −10–0 °C significantly lower than that in the range of 0–10 °C. It shows that the asphalt mixture is elastic at low temperatures, but with a rise in temperature, the asphalt mixture becomes gradually viscous and ductility is improved, so the flexural tensile strain increases. Taking 0 °C as the characteristic point for analysis, compared with the original asphalt mixture, the *ε*_B_ values of wood tar-based rejuvenated asphalt mixture and RA-102 rejuvenated asphalt mixture decreased by 4.38 and 7.19% respectively, and the *S*_B_ values increased by 0.83 and 2.65%, respectively, demonstrating that the low-temperature crack resistance of the wood tar-based rejuvenated asphalt mixture was slightly lower than that of the original asphalt mixture and higher than that of the RA-102 rejuvenated asphalt mixture, and the deformation recovery ability of the wood tar-based rejuvenator to aged asphalt was better than that of the RA-102 rejuvenator. The reason for this was that the addition of wood tar, biomass fiber, and plasticizer in wood tar-based rejuvenated asphalt improved the fluidity and ductility of the asphalt mixture, forms fiber skeleton network [22], strengthens the crosslinking between asphalt and aggregates, and improved the deformation performance and toughness of the mixture.

### 3.4. Trabecular Bending Creep Test

**Figure 9:**
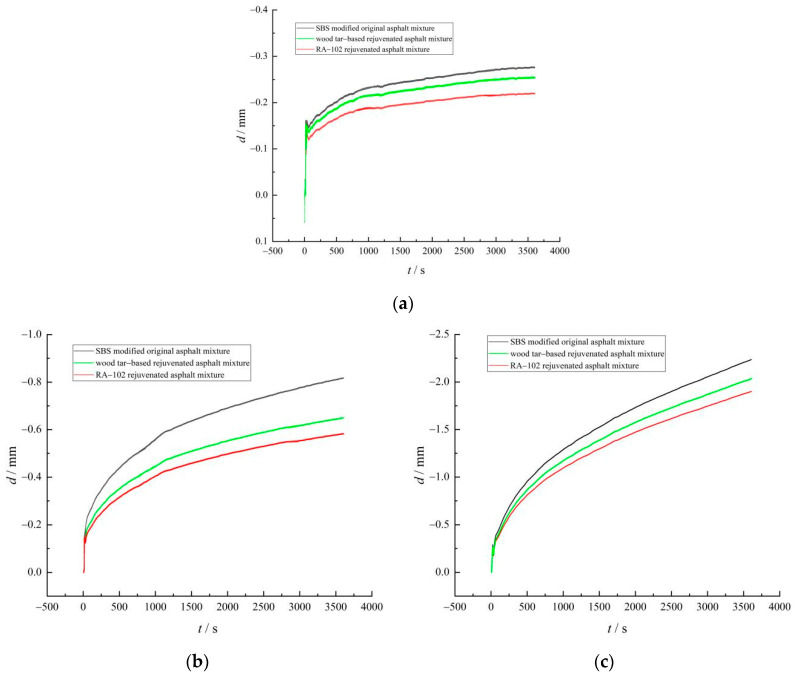
Creep curves of each asphalt mixture. (**a**) Creep curve at −10 °C, (**b**) creep curve at 0 °C. and (**c**) creep curve at 10 °C.

The creep curves of each asphalt mixture are shown at different temperatures in Figure 9.

As shown in Figure 9, the creep strain of the three asphalt mixtures decreased with a decrease in temperature, showing that as the temperature decreases, the mixture gradually “hardens, and the viscous component in the mixture changes to an elastic component [23]. The temperature stress cannot be eliminated due to deformation, resulting in the deterioration of low-temperature performance or even cracking of the asphalt mixture. With the gradual decrease in temperature, the deflection curve of each asphalt mixture tends to be stable, and there is no obvious last stage of creep damage (failure stage) [24]. The reason is that the decrease in temperature leads to the brittleness of asphalt and the continuous reduction in the proportion of viscous components, resulting in predominant elastic deformation. The destruction form of asphalt at low temperatures is close to elastic deformation, which decreases the flexibility and deformation ability of the mixture at low temperatures.

As shown in Figure 9a, the original asphalt mixture enters the creep stability period around 1500 s, whereas the wood tar-based rejuvenated asphalt mixture and RA-102 rejuvenated asphalt mixture enter the creep stability period around the 1750 s and 1850 s, respectively. Both rejuvenated asphalt mixtures enter the creep stability period later than the original asphalt mixture, but the wood tar-based rejuvenated asphalt mixture enters the creep stability period earlier than the RA-102 rejuvenated asphalt, and its creep strain is greater than that of RA-102 rejuvenated asphalt mixture. This suggests that wood tar-based rejuvenator can improve the low-temperature performance of aged asphalt mixture, and wood tar-based rejuvenated asphalt mixture has good creep deformation ability at low temperatures. When the temperature stress caused by the sudden drop in temperature acts on the asphalt mixture, the wood tar-based rejuvenated asphalt mixture can reduce temperature stress, thus reducing the risk of cracking in the asphalt mixture.

### 3.5. Creep Damage Model

**Figure 10:**
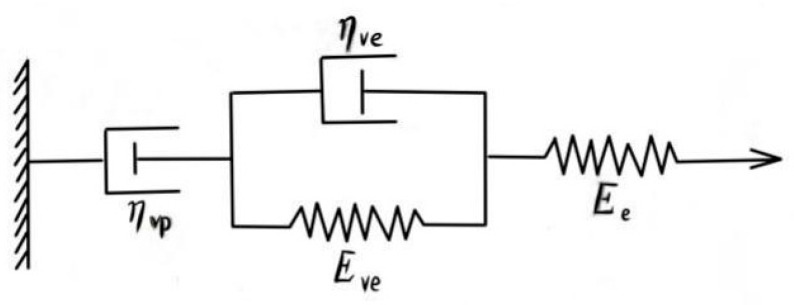
Improved Burgers model.

Burgers model is a four-component model composed of the spring element and sticky element in series. This model can effectively explain the mechanical behavior of viscoelastic material, such as creep recovery and stress relaxation, so it can be used to study the low-temperature crack resistance of asphalt mixtures. At low temperatures, there was not only plastic hardening, but also damage softening during the creep process of asphalt mixture [25]. Both exist simultaneously and compete with each other. When plastic hardening is dominant, creep presents at a weak creep stage; when damage softening is dominant, creep presents at accelerated creep stages, so the creep deformation characteristics of the asphalt mixture cannot only be described by considering a single factor. In order to comprehensively consider the influence of the two factors on the creep deformation characteristics of the asphalt mixture, the rheological method and damage mechanics were adopted to improve the Burgers model (as shown in Figure 10) [26]. The creep model-dependent damage softening was obtained as follows.

The total strain of the asphalt mixture *ε* can be expressed as [26]:

(2) $$\varepsilon =\varepsilon_{e}+\varepsilon_{ve}+\varepsilon_{vp}$$

(3) $$\varepsilon_{e}=\frac{\sigma }{E_{e}}$$

(4) $$\varepsilon_{ve}=\frac{\sigma }{E_{ve}}\left(1-e^{\frac{-E_{ve}t}{\eta_{ve}}}\right)\varepsilon_{ve}=\frac{\sigma }{E_{ve}}\left(1-e^{\frac{-E_{ve}t}{\eta_{ve}}}\right)$$

The viscoplastic strain rate can be expressed as [26]:

(5) $$\dot{\varepsilon_{vp}}=\frac{B\sigma^{n}}{\eta_{vp}}=\frac{B\sigma^{n}}{\;A\varepsilon_{vp}^{p}}$$

Under constant stress $\sigma_{0}$, viscoplastic strain is obtained as [26]:

(6) $$\varepsilon_{vp}={\left[\frac{B}{A}\left(p+1\right)\right]}^{\frac{1}{p+1}}\sigma_{0}^{\frac{n}{p+1}}t^{\frac{1}{\;p+1}}$$

where *ε_e_* is instantaneous elastic strain, *ε_ve_* is viscoelastic strain, *ε_vp_* is viscoplastic strain, *σ* is loading stress (MPa), *E_e_* is the elastic modulus (MPa), *E_ve_* is the elastic modulus of Kelvin model (MPa), $\eta_{ve}$ is the viscosity coefficient of the Kelvin model, $\eta_{vp}$ is the viscosity coefficient of viscoplastic elements, and *A*, *B*, *p*, and *n* are material parameters.

Under external loads, material damage leads to reduction the effective area of material. The effective stress of material under external load is [26]:

(7) $$\bar{\sigma }=\frac{\sigma }{1-D}$$

where $\bar{\sigma }$ is effective stress (Mpa), *D* is damage factor, and 0 ≤ *D* ≤ 1.

For a uniaxial compression stress state, the Kachanov creep damage model can be adopted [26]:

(8) $$D=C\sigma^{V}{\left(1-D\right)}^{-V}$$

where D is the change rate of damage factor and *C* and *v* are the material constants related to temperature.

The critical failure time of creep damage is obtained by integrating Equation (8) [26]:

(9) $$t_{R}={\left[C\left(v+1\right)\sigma^{v}\right]}^{-1}$$

Thus, the damage factor *D* is obtained [26]:

(10) $$D=1-{\left(1-\frac{t}{t_{R}}\right)}^{\frac{1}{v+1}}$$

Combined with the total strain equation, the creep damage model of asphalt mixture under constant stress considering viscoplasticity can be obtained as [26]:

(11) $$\varepsilon =\frac{\sigma_{0}}{{\left(1-\frac{t}{t_{R}}\right)}^{\frac{1}{v+1}}\;E_{e}}+\frac{\sigma_{0}}{{\left(1-\frac{t}{t_{R}}\right)}^{\frac{1}{v+1}}E_{e}}\left(1-e^{-\frac{E_{ve}t}{\eta_{ve}}}\right)+{\left[\frac{B}{A}\left(p+1\right)\right]}^{\frac{1}{p+1}}\sigma_{0}^{\frac{n}{p+1}}t^{\frac{1}{\;p+1}}$$

**Figure 11:**
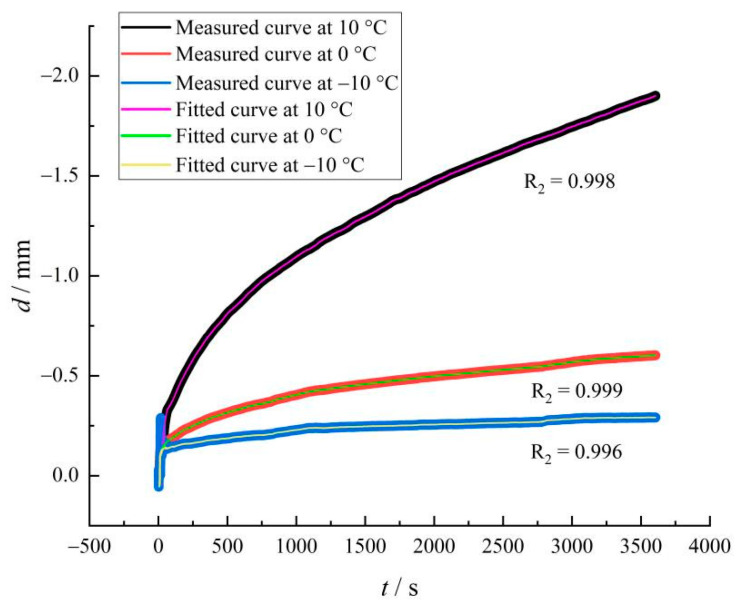
Measured displacement and fitted curve of wood tar-based rejuvenated asphalt mixture.

Origin software was used to fit and analyze the flexural creep test results of the wood tar-based rejuvenated asphalt mixture at different temperatures to verify the rationality of the creep damage model of asphalt mixtures, considering viscoplasticity at low temperatures. The fitted results are shown in Figure 11.

It can be seen from Figure 10 that the fitted curve of the creep damage model fit well with the measured curve, and had a high correlation, indicating that the creep damage model could describe the flexural creep performance of the wood tar-based rejuvenated asphalt mixture well at low temperatures, and the deformation characteristics of other low temperatures could be inferred from this model.

## 4. Conclusions

(1) Compared with the original asphalt, wood tar-based rejuvenated asphalt had lower creep stiffness and higher creep slope. The low temperature classification of wood tar-based rejuvenated asphalt was basically in the same grade as original asphalt and RA-102 rejuvenated asphalt.
(2) The low-temperature crack resistance of the wood tar-based rejuvenated asphalt mixture was obviously better than that of the RA-102 rejuvenated asphalt mixture, but slightly worse than the original asphalt mixture. After combining wood tar and biomass fiber, wood tar can fully permeate the new asphalt, effectively alleviating the bond failures between asphalt and aggregates and improving the stiffness of the mixture, so that the toughness and crack resistance of the rejuvenated asphalt mixture at low temperatures are improved.
(3) The established creep damage model could better describe the flexural creep performance of rejuvenated asphalt mixtures at low temperatures, and could be used to infer the deformation characteristics at other temperatures.
(4) The creep damage model established in this study could be effectively extended to the study of low-temperature crack resistance of other types of recycled asphalt mixtures. Due to the wide source of raw materials and green environmental protection, wood tar-based rejuvenator has big potential to be promoted and applied in this way, which could effectively replace the extraction oil rejuvenator.
(5) In later stages, we plan to analyze the low-temperature cracking resistance of the reclaimed asphalt mixture from the mechanism of wood tar-based regenerating agents on aging asphalt at the micro-nano level.

## Figures and Tables

**Table 1:** Basic properties of each asphalt [13].

Asphalt Type	Penetration (25 °C)/0.1 mm	Penetration Index	Ductility (15 °C)/cm	Viscosity (135 °C)/Pa·s	Softening Point /°C
SBS-modified original asphalt	66.1	−1.136	117	0.34	48
RA-102 rejuvenated asphalt	63.3	−0.768	113	0.45	55
Wood tar-based rejuvenated asphalt	63.5	−0.870	102	0.47	57

**Table 2:** Basic properties of wood tar and biomass fiber.

Material Type	Moisture Content/%	Density/(g·ml^−1^)	Relative Density	pH	Length/μm
Wood tar	4.2	1.15	--	2.14	--
Biomass fiber	<3.0	--	0.91–0.95	--	400–2000

**Table 3:** Basic properties of each rejuvenator [13].

Technical Index	RA-102 Rejuvenator	Wood Tra-Based Rejuvenator	Specified Value
Viscosity/(60 °C, Pa·s)	5370	4202	50–60,000
Flash point/°C	241	213	≥220
Saturates content/%	20.3	21.4	≤30
Aromatics content/%	64.2	34.2	--
Viscosity ratio before and after RTFOT *	1.3	1.4	≤3
Mass change before and after RTFOT/%	0.5	0.3	[−3, 3]

* Note: Rolling thin film oven test.

**Table 4:** Basic technical indexes of coarse aggregate [13].

Technical Index		Test Value	Specified Value
Crushing value/%		9.4	≤20
Los Angeles abrasion/%		10.7	≤24
Polishing value/%		50	≥42
Apparent specific gravity	10–16 mm	2.8053	≥2.6
	5–10 mm	2.8265	
Water absorption/%	10–16 mm	0.458	≤2.0
	5–10 mm	0.679	
Percent of flat and elongated particles/%	10–16 mm	4.5	≤10
	5–10 mm	10.8	≤15

**Table 5:** Basic technical indexes of fine aggregate [13].

Technical Index	Apparent Specific Density	Sand Equivalent/%	Angularity/s
Test value	2.7404	71	50
Specified value	≥2.5	≥60	≥40

**Table 6:** Basic technical indexes of mineral powder [13].

Technical Index	Apparent Specific Density	Water Content/%	Plasticity Index/%	Hydrophilic Coefficient
Test value	2.714	0.4	2	0.6
Specified value	≥2.5	≤1	<4	<1

**Table 7:** SCB test results of each asphalt mixture.

Test Index	SBS-Modified Original Asphalt	Wood Tar-Based Rejuvenated Asphalt	RA-102 Rejuvenated Asphalt
Flexural tensile stress σB/MPa	3.142	2.946	2.565
Flexural tensile strain εB/µε	2143.7	2018.4	1733.6
Fracture energy density $\frac{dw}{dv}$/kPa	0.038	0.041	0.034
